# Estimation of Adult Sex Ratio and Size-Related Sexual Dimorphism Based on Molecular Sex Determination in the Vulnerable La Selle Thrush, *Turdus swalesi*

**DOI:** 10.3390/ani14060842

**Published:** 2024-03-08

**Authors:** Jean-Marry Exantus, Etienne Bezault, Christopher Cambrone, Frank Cézilly

**Affiliations:** 1 Caribaea Initiative, c/o Université des Antilles, Campus de Fouillole, F-97110 Pointe-à-Pitre, CEDEX, France; etienne.bezault@univ-antilles.fr (E.B.); christopher.cambrone@caribaea.org (C.C.); frank.cezilly@u-bourgogne.fr (F.C.); 2 UMR BOREA, CNRS 8067, MNHN, IRD, Sorbonne Université, Université Caen Normandie, Université des Antilles, Campus de Fouillole, F-97110 Pointe-à-Pitre, CEDEX, France

**Keywords:** CHD-1 gene, conservation, Haiti, Hispaniola, sex ratio, tail length, *Turdus swalesi*

## Abstract

**Simple Summary:**

This study assessed the adult sex ratio and sexual dimorphism in body size in the vulnerable La Selle Thrush, *Turdus swalesi*, over a 26-month period, in Haiti. We found that the overall adult sex ratio was significantly male-biased. However, this was only true during the non-breeding season, suggesting that males may show more site tenacity than females, although true differences in adult survival between males and females cannot be ruled out. We observed a slight, albeit significant, sexual dimorphism in size, with males having, on average, both a longer wing chord and a longer tail than females. Tail length was the best predictor of sex in a logistic regression model, with about 80% of individuals being correctly assigned to their actual sex. Understanding the causes and consequences of the observed male-biased sex ratio might be important for the conservation of the La Selle Thrush, particularly in the face of ongoing severe deforestation in Haiti.

**Abstract:**

Sex-determination is of particular importance in avian ecology and conservation. However, many bird species show no conspicuous sexual dimorphism, such as the La Selle Thrush, *Turdus swalesi*, a vulnerable species endemic to Hispaniola. We captured individuals in southeastern Haiti, in 2019–2022. For each one, we collected contour feathers or blood samples for molecular sex identification based on the CHD-1 gene. In addition, we took body measurements of several captured individuals and recorded their weight. Out of a total of 65 birds, 45 were identified as males and 20 as females, indicative of a significantly male-biased sex ratio. However, analyses of first captures showed that the sex ratio at our study site was male-biased only outside of the breeding season, suggesting that females may disperse at that time while males remain on their territories. Sexual dimorphism was limited to wing chord length and tail length, with males being larger than females. Tail length was the best predictor of sex in a logistic regression model and correctly classified about 80% of individuals as male or female. We discuss our results in relation to previous studies of sex ratio and sexual dimorphism in turdid species and address their relevance for the conservation of avian species in one of the major protected forest areas in Haiti.

## 1. Introduction

Although sexual dimorphism can be particularly conspicuous in some bird species, particularly in polygynous ones [1], it can be quite subtle in others, making the distinction between males and females through direct visual observation particularly difficult, if not impossible. Sex identification of sexually monomorphic bird species is, however, of particular importance to understand their evolutionary ecology [2], investigate sex-related differences in behavior [3,4], estimate demographic parameters [5,6], and is often relevant to conservation and management [7,8,9]. In particular, sex identification is crucial for the estimation of the adult sex ratio (the proportion of males and females in the adult population) in monomorphic bird species. The adult sex ratio is a demographic trait of a population that depends on sex ratios at conception and birth, and is then modified by sex differences in rates of maturation, mortality, dispersal, and immigration [10]. The adult sex ratio is an important characteristic of animal populations [11,12], and has direct relevance to behavioral ecology, evolution, and conservation [13,14]. In particular, deviations from a balanced adult sex ratio (i.e., an equal proportion of males and females in the adult population), can have negative consequences for individual fitness and population viability in birds [15,16].

Various methods have been used to provide accurate sexual determination in monomorphic avian species. Whereas earlier studies relied on anatomical investigations [17] or sex-specific behaviors [18,19], the advent of molecular techniques has provided a standardized and reliable method to sex monomorphic bird species based on sex chromosome-specific markers [20,21]. Avian DNA can indeed be obtained relatively easily from non-invasive (eggshell swab, shed feather, and feces), nondestructive (plucked feather and buccal swab), or invasive (blood) methods [22,23,24,25,26,27]. Molecular sexing is now routinely used to assess the extent of sexual dimorphism in body size and/or variation in sex ratio in monomorphic avian species [28,29,30,31,32,33,34]. 

The La Selle Thrush, *Turdus swalesi*, is a sexually monomorphic passerine endemic from Hispaniola. This large-bodied turdid species has black, rufous, and white plumage, and a yellow to orange bill and eye-ring [35]. The species was originally described about one century ago from the “Massif de La Selle”, in southeastern Haiti [36]. It was only known from there until individuals were observed in the Sierra de Bahoruco (which is the eastern extension of the La Selle ridge into the Dominican Republic) in 1971, and then in the Sierra de Neiba and the Cordillera Central in 1975 [37,38]. Based on lighter back plumage, populations from the Sierra de Neiba and Cordillera Central were later considered as distinct subspecies, T. *swalesi dodae* [39]. Although the species is currently classified as vulnerable in the IUCN Red List [40], very limited information is available about its ecology, behavior, and population dynamics. The species is at risk, given its restricted distribution area, and is threatened by habitat loss and fragmentation, particularly due to intense deforestation in Haiti [41,42], and, possibly, exotic mammal predators (J.M. Exantus unpubl. data). 

Here, we report new and original data on the La Selle Thrush, collected as part of a long-term capture-mark-recapture program of the La Selle ridge population in Haiti. Our objective was to assess the adult sex ratio and the extent of sexual dimorphism in adult body size in this poorly studied species. To that end, we captured and measured individuals from which we collected feathers or blood samples as sources of DNA. We then used the accurate DNA-based test method, PCR amplification, to sex the captured individuals [21,43,44,45]. Based on previous evidence for seasonal variation in the sex ratio of turdid species in relation to breeding activity in both tropical and temperate areas [46,47], we expected differences in the adult sex ratio between the breeding and non-breeding seasons. We discuss our results in relation to variation in both sex ratio and the extent of sexual dimorphism in natural populations of other turdid species, and to conservation issues. 

## 2. Materials and Methods

### 2.1. Study Site and Data Collection

**Figure 1:**
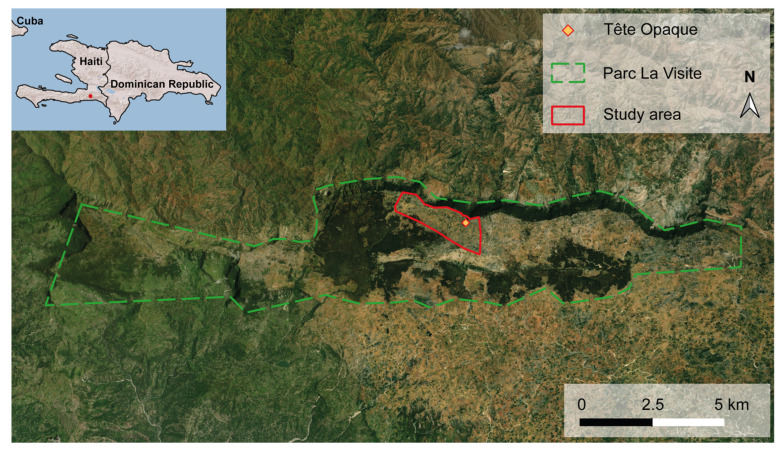
Location of study area “Tête Opaque” within the limits of Parc La Visite, southeastern Haiti. © 2023 Microsoft Corporation, accessed on 24 July 2023.

Mist-netting of La Selle Thrushes took place in the locality of “Tête Opaque” (18°20.928′ N, 072°14.347′ W), within the Parc La Visite (Figure 1), located in the La Selle ridge, at an altitude varying between 2146 and 2260 m. The area is located to the north of the first municipal district of Baie d’Orange, commune of Belle Anse, in Haiti’s Southeastern district. 

We carried out 13 consecutive field surveys at our field station in Parc la Visite between December 2019 and January 2022. The duration of field sessions varied from 3 to 15 days (median = 12.5), partly depending on weather conditions, while the duration of intervals between consecutive field sessions varied between 20 and 68 days (median = 40), except for an interruption from 26 February to 26 September in 2020 due to the COVID pandemic and safety issues in Haiti. The breeding season of the La Selle Thrush lasts from April to the end of July [48,49,50], J.M. Exantus pers. obs., such that three field sessions took place during the 2021 breeding season, whereas all other sessions took place outside of the breeding season. 

On each session, we used footpaths varying between 800 and 1000 m in length and pre-existing openings in vegetation (abandoned agricultural plots) created by humans to deploy mist nets (Ecotone^®^, Gdynia, Poland, 6 × 3 m, 19 mm mesh, most suitable for the La Selle Trush and Ecotone ^®^ 12 × 3 m, 30 mm mesh) at regular intervals. The total length of mist nets varied progressively from 12 m to 96 m from the first to the last session. The position of mist nets varied between sessions depending on interferences with agricultural activities by local people, such that the overall study area covered about 268 ha (see Figure 1), as determined by GPS (Garmin^®^, Olathe, KS, U.S.A., Montana 680) coordinates. We regularly broadcasted the call of the la Selle Thrush to attract individuals to the mist nets and increase capture rates [51]. Mist nets were checked every 25 minutes and on hearing birds screaming loudly after being captured, or to ward off free-ranging animals (cattle, sheep, horses, and pigs) that ventured too close to the mist nets. In case of rain or strong wind, we temporarily closed or did not operate the mist nets. 

Each captured individual was ringed with a unique combination of an aluminum leg band engraved with an alphanumeric reference code and 1–2 colored plastic rings. We measured tarsus length (TarL), tarsus width (TarW), bill width at nostrils (BWN), bill width at the tip (BWT), bill length to the base of the skull (BL), and head length including bill (HBL) using a dial caliper with a 0.1 mm accuracy. In addition, we measured wing chord length (WCL) and tail length (TaiL) to the nearest millimeter, using a metal ruler and a flat ruler with a stop at zero, respectively. We measured the weight (W) of individuals to the nearest gram using a Pesola 500 g spring scale. All measurements were taken according to recommendations provided by the Centre de Recherche sur la Biologie des Populations d’Oiseaux [52] and the North American Banding Council [53].

We collected two different tissues as their effectiveness as source of high-quality DNA for molecular analyses may vary between bird species [26]. We first used a non-destructive method relying on contour feather sampling [22,23,24,25]. We collected three to five small contour feathers from each captured individual and placed them in envelopes with “silica gel” moisture absorber for dry storage. Using a more invasive method, we collected ~15 μL blood samples from the brachial vein from a subsample of individuals [54,55]. The collected blood samples were preserved in ethyl alcohol of 99.99% volume purity. Blood samples were stored in the laboratory at −20 °C for 18 months until DNA extraction [26].

The entire handling process of each captured bird, including blood and/or feather sampling and, when time allowed, biometric measurements, took an average of 13 minutes per bird. Complete processing was not possible for birds that were captured late in the afternoon. Such birds were quickly released immediately after ringing and feather collection, without taking biometric measurements or collecting a blood sample. 

### 2.2. Molecular Analyses

Following the protocol developed by Cambrone et al. [26], we used a commercial DNeasy Blood and tissue kit (Qiagen Inc., Valencia, CA, USA) to extract DNA from blood samples. For DNA extraction from collected feather samples, we used calamus tips and blood clots and/or barbs from feathers [56,57]. Samples were then digested overnight by adding 30 μL of 1M dithiothreitol (DTT) to the commercial kit lysis solution, including proteinase K [58]. The same PCR (polymerase chain reaction) protocol was used for DNA samples from blood and feathers to sex individuals using primers developed by Fridolfsson & Ellegren [59], while adding 1.50 μL of Bovine Serum Albumin, following the PCR protocol developed by Cambrone et al. [26]. We identified sex using size variation of introns of the Chromo-Helicase-DNA binding protein genes (CHD1-Z and CHD1-W), using 2550F/2718R primer pair, standard PCR conditions, and agarose gel (3%) electrophoresis [26,59]. Intron size variation was easily scored, with CHD1-W = 450 pb and CHD1-Z = 750 pb, using a 100 pb ladder (Nippon Genetics Co., Düren, Germany). For a few individuals for which sex identification was ambiguous on the first PCR, we conducted two additional PCR and electrophoresis. 

### 2.3. Statistical Analysis

We first assessed deviation from a balanced adult sex ratio among all sexed individuals using a binomial test. In addition, we compared the relative proportions of first-captured males and females during the breeding and non-breeding seasons using a Fisher’s exact test. 

We relied on Shapiro–Wilk tests to assess the normality of biometric measurements. As most of them were not normally distributed, we used non-parametric tests to assess correlations between biometric measurements and differences between sexes. We then excluded measurements that were significantly correlated between themselves (*p* < 0.05) to build logistic regression models and predict the sex of individuals. We favored the use of logistic regressions over a discriminant analysis given the relatively small sample size [60].

We compared the fit of logistic regression models using difference in the small-sample corrected Akaike Information Criterion, AICc, and considered models with Δ AICc < 4 as equally informative [61]. We analyzed all data using the package “stats” in the R software, version 4.2.2 [62], and considered the results significant at the 0.05 level.

## 3. Results

Overall, we obtained blood from 45 individuals and relied on feathers as a source of DNA for 21 additional birds. DNA extraction was successful for all individuals, but one for which we failed to extract DNA of high enough quality from contour feathers. Out of the 65 remaining individuals, 45 were identified as males and 20 as females (binomial test, *p* = 0.0013). The proportion of individuals identified as males or females was independent of the tissue (blood of feathers) that was used as a source of DNA (Chi-square test, X^2^ = 0.462, d.f. = 1, *p* = 0.4968). However, there was a significant difference in the proportions of first-captured males and females between the breeding and the non-breeding seasons (Table 1; Fisher’s exact test, *p* = 0.0034).

### Sexual Size Differences in the La Selle Thrush

Biometric measurements were available for 43 individuals of known sex (29 males and 14 females). Only tarsus length and weight were normally distributed (Shapiro–Wilk test, *W* = 0.98, *p* = 0.4887 and *W* = 0.96, *p* = 0.1723, respectively), whereas all other measured traits did not follow a normal distribution (0.76 ≤ *W* ≤ 0.94, 0.0289 ≤ *p* ≤ 0.0001). Table 2 shows values for biometric traits according to assigned sex and corresponding non-parametric statistical tests for significant differences. Males and females differed significantly only for wing chord length and tail length. However, wing chord length and tail length were positively correlated among individuals (Spearman rank correlation coefficient, *r*_s_ = 0.44, n = 43, *p* = 0.0035). 

We, therefore, used them separately to predict the sex of individuals in logistic regression models with sex as a binomial variable. Table 3 shows the four best models retained by model selection. In terms of accuracy of sex assignment, the best model with only tail length correctly classified 81.4% of individuals, with no difference between sexes (Fisher’s exact test on proportions of correctly and incorrectly classified individuals according to sex, *p* = 0.4038). Based on this model, the probability that a bird is a male can be calculated following the following equation: *p* (male) = 1/1 + e ^−(−23+0.23 (tail length))^, with 0 ≤ *p* (male) ≤ 1. The higher the *p* (male) value is, the more likely the individual is a male. The second-best model including tarsus width in addition to tail length provided exactly the same percentage of correct sex assignment. 

## 4. Discussion

Previous studies on other turdid species relied exclusively on blood samples to obtain DNA for molecular sexing [11,46,47,63,64,65]. Here, we used both blood and contour feathers with equal success to assign a sex to individuals. We found no significant difference in the sex ratio of birds sexed with DNA extracted from feathers or blood. As plucking of contour feathers might be regarded as a less intrusive and less harmful method to obtain DNA [25,66], we recommend giving priority to this technique when biological samples are only needed for sex determination, especially for passerine species of conservation interest. 

The strongly male-biased overall adult sex ratio observed in our study population deserves particular attention. On the one hand, it might be a direct consequence of using playback around mist nets to increase capture efficiency, as previously advanced to explain male-biased sex ratio among captured individuals of another Caribbean-endemic thrush species [67]. Indeed, males of some passerine species tend to be more strongly attracted by playbacks than females [68]. On the other hand, Chin et al. [69] found no difference between sexes in attraction towards audio lures in wintering Wood Thrushes, *Hylocichla mustelina*. In addition, we captured or recaptured significantly more male individuals than females during the non-breeding season, whereas, although the sample size was limited, there was no difference in the proportions of male and female La Selle Thrushes during the breeding season. This seasonal pattern of variation in adult sex ratio is similar to what was reported by Ritter et al. [46] for the White-necked Thrush, *Turdus albicollis*, in southeastern Brazil. One possibility, then, is that the two sexes differ in their strategy outside of the breeding season with males staying on their territories while females disperse out of the area [70]. Males may benefit from remaining on their territories to maintain ownership, whereas females may benefit from dispersing in various ways. First, seasonal variation in male–female competition for food resources may favor female dispersal if males are the most competitive sex, particularly during the dry season when food availability is reduced [71,72]. Second, females of monogamous bird species may disperse during the non-breeding season to assess alternative breeding opportunities at other sites [73,74]. Radio-tracking of individuals of both sexes (see Aubry et al., [75]) might be useful in the future to document seasonal variation in movements and home ranges of the species. Third, a higher proportion of males than females has been previously reported in several tropical *Turdus* species [11,46,64,76]. The overall male-biased adult sex ratio observed in the present study could thus be a particular feature of the *Turdus* species in tropical forests if, for instance, adult survival rate tends to differ between the two sexes [70,77]. Such differences in adult survival between the sexes might be related to the estimation of sex-specific adult survival in the La Selle Thrush using capture-mark-recapture data [78], and further investigations are thus needed to better understand variation in the adult sex ratio of this vulnerable species. 

Overall, body size and weight overlapped largely between male and female La Selle Thrushes. However, we evidenced a subtle, albeit significant, sexual dimorphism in body size in the La Selle Thrush, with median tail length being about 10% longer in males compared to females, and median wing length being about 3.5% longer, with both characters being positively correlated between themselves. Similarly, several studies found that wing length was sexually dimorphic and longer in males of thrush species [11,46,47,63,64,65,79], whereas tail length was seldom included in the analysis of sexual dimorphism in turdid species (however, see Frey et al. [79]). Although direct evidence is lacking for the La Selle Thrush, wing flicking and tail raising have been observed during ritualized aggressive encounters in other thrush species [80,81], such that the observed sexual dimorphism may indicate potential male social dominance over females. 

The percentage of correct sex assignment using a regression logistic model based on tail length was about 80% and compares with other studies of turdid species using logistic regression models or discriminant analysis to determine sex from morphometric measurements [47,65]. However, sex identification of La Selle Thrushes in the field solely based on morphometrics appears poorly reliable, with an error rate of about 20%, thus justifying the use of molecular tools to ascertain the sex of individuals, particularly for future studies investigating demographic parameters and behavior. 

## 5. Conclusions

Our study provides new perspectives for a long-term survey of the vulnerable La Selle Thrush. First, the collection of contour feathers appears to be a reliable method to obtain DNA of enough quality for molecular sexing. Future investigations should check to what extent DNA extracted from feathers can also be used for the analysis of genetic structure, using both mitochondrial and nuclear markers. This is of prime importance to assess the overall degree of genetic diversity at the species level and assess the actual extent of genetic differentiation between the two recognized subspecies. Second, the causes and consequences of the observed male-biased sex ratio should be addressed using a larger sample of individuals covering a larger part of the species distribution area. In particular, capture-mark-recapture analyses may prove useful in estimating potential sex-related differences in survival and probability of capture in pre-fledging, juvenile, and adult individuals [6,82]. 

Continued monitoring of the La Selle ridge population of the La Selle Thrush is also of high importance for avian conservation in Haiti as the species demographic trends might be representative of that of several other forest-dependent avian species known to occur in Parc La Visite, including several endemic ones [83,84], in the face of persistent environmental degradation. In that respect, assessing variation in the adult sex ratio among local avian species in relation to their degree of forest dependency might provide a clue to the consequences of deforestation for local bird species, as deviation from a balanced sex ratio tends to be more severe in populations of threatened avian species than in non-threatened ones [85]. Although the ability to conduct ornithological research in Haiti remains currently limited due to political instability, lack of local scientific expertise, and limited access to equipment and funding [86], the present study and a few recent ones [87,88] raise some hope for the future.

## Figures and Tables

**Table 1:** Proportions of first-captured males and females according to season.

	Males	Females	Percentage of Females
Breeding season	2	7	77.78%
Non-breeding season	43	14	24.56%

**Table 2:** Median values, interquartile range (IQR), minimal (Min), and maximum (Max) values of body size (mm) and weight (g) according to assigned sex and corresponding statistical tests (Mann–Whitney test for two independent samples). Significant differences are highlighted in bold.

	Males (n = 29)				Females (n = 14)					
	Median Value	IQR	Min	Max	Median Value	IQR	Min	Max	z	*p*
TarL	46.3	44.3–48.0	40.3	52.4	46.1	43.8–49.3	42	55.5	0.29	0.7755
TarW	33	33.0–33.6	31	34	33	33.0–33.0	31	34	1.62	0.1031
BWN	7.2	6.8–8.0	5.4	10.4	7.4	6.9–7.9	6.1	8.5	0.27	0.7849
BWT	2.2	2.0–2.4	2	2.9	2.2	2.1–2.4	2	2.9	0.52	0.606
BL	21.8	20.2–23.4	18.6	27	22.4	20.1–23.9	19.5	27.3	0.43	0.6686
HBL	56.9	55.1–58.5	50	60	57	55.5–58.7	50.5	61.4	0.31	0.7557
WCL	128	126–130	120	132	124	120–130	120	130	2.38	**0.0173**
TaiL	110	105–110	99	120	100	98–109	90	110	3.11	**0.0019**
W	120	110–125	98	135	120	110–120	90	150	0.36	0.707

**Table 3:** Parameters of the best four logistic regression models showing the association between body measurements and sex in the La Selle Thrush (n = 43). Response variable was the sex of individuals and explanatory variables were four different combinations of measurements.

	Goodness of Fit		Model Selection	
Independent variables	X^2^	*p*	AICc	Δ _AICc_
Tail length	12.15	0.0005	46.42	-
Tail length and tarsus width	14.07	0.0009	46.81	0.41
Wing chord length and tarsus width	10.64	0.0049	50.24	3.82
Wing chord length	7.16	0.0075	51.41	4.99

## Data Availability

The data that support the findings of this study are available from the corresponding author, [JME], upon reasonable request.
